# Lnc5q21.2, a novel long intergenic RNA, sensitizes colorectal cancer cells to ATR inhibitor by activating Wnt pathway

**DOI:** 10.1515/jtim-2025-0040

**Published:** 2025-10-16

**Authors:** Meiying Zhang, Cheng Zhu, Aiai Gao, James G. Herman, François Fuks, Jianjun Luo, Xiaomo Su, Hengmi Cui, Runsheng Chen, Mingzhou Guo

**Affiliations:** Department of Gastroenterology & Hepatology, the First Medical Center, Chinese PLA General Hospital, Beijing, China; Medical College of NanKai University, Tianjin, China; The Hillman Cancer Center, University of Pittsburgh Cancer Institute, Pittsburgh, PA, USA; Laboratory of Cancer Epigenetics, Free University of Brussels (U. L.B.), Brussels, Belgium; Key Laboratory of RNA Biology, Institute of Biophysics, Chinese Academy of Sciences, Beijing, China; Institute of Epigenetics and Epigenomics and College of Animal Science and Technology, Yangzhou University, Yangzhou, Jiangsu Province, China

**Keywords:** Lnc5q21.2, DNA damage repair, ATR, Wnt signaling, colorectal cancer

## Abstract

**Background and objectives:**

Colorectal cancer (CRC) is still the leading cause of cancer-related death. With the recognizing the importance of long non-coding RNA (LncRNA) in development and cancer, it is urgently to identify new LncRNA and understand the mechanism to develop novel therapeutic strategies.

**Methods:**

Nine CRC cell lines, 52,146 and 285 cases of normal colorectal mucosa, adenoma and CRC samples were utilized. Northern blot, rapid amplification of cloned cDNA ends, RNA pulldown, Mass spectrum, RNA immunoprecipitation, CRISPR/Cas9, fluorescence *in situ* hybridization assays and xenograft mice model were employed.

**Results:**

Lnc5q21.2 is identified to be a novel long intergenic non-coding RNA and its full length is 668 nt. Lnc5q21.2 is mainly located in cell nucleus and its expression is regulated by N^6^-methyladenine (6mA) modification. Compared to adjacent tissue, the levels of Lnc5q21.2 were increased significantly in CRC samples (*P* < 0.001), with a progression tendency from noncancerous colorectal mucosa, adjacent tissue, adenoma to CRC samples (*P* < 0.001). Lnc5q21.2 highly expression is associated with alcohol consumption and poor prognosis (both *P* < 0.05). Lnc5q21.2 promotes cell proliferation, migration, invasion and cell cycle progression. Lnc5q21.2 activates Wnt signaling by interacting with homeobox A10 (HOXA10) and down regulating empty spiracles homeobox 2 expression. Further study demonstrates that Lnc5q21.2 promotes DNA damage repair by enhancing ATR signaling. Lnc5q21.2 sensitizes CRC cells to ATR inhibitor both *in vitro* and *in vivo*.

**Conclusions:**

Lnc5q21.2 is a novel lncRNA. Lnc5q21.2 promotes ATR pathway by activating Wnt signaling via interacting with HOXA10. Lnc5q21.2 sensitizes CRC cells to ATR inhibitor both *in vitro* and *in vivo*.

## Introduction

Colorectal cancer (CRC) is one of the major causes of cancer-related death.^[1]^ The 5-year survival rate is 91% for localized CRC patients and 14% for metastatic disease.^[2]^ The discovery of regulatory elements is becoming the primary part of cancer biology research in the postgenomic era.^[3]^ Long noncoding RNAs (LncRNA) have emerged as important modulators in cancer initiation and development.^[4]^ Recently, lncRNAs were recognized to play a determining role in cellular phenotype during development.^[5]^ LncRNAs have been linked to chromatin remodeling, DNA damage repair, necroptosis and drug resistance in cancer and other diseases.^[6, 7, 8, 9]^ More than 70% of the human genome may yield noncoding RNA transcripts.^[4,10]^ With the development of new techniques, more important lncRNA fragments related to diseases, tissue structure or cell type specificity will be discovered. Using spatial transcriptomics, a batch of lncRNA fragments were found linked to CRC metastatic tissues.^[11,12]^ However, very limited numbers of lncRNAs were identified with full-length sequences. Deep understanding the function of lncRNAs and their regulation network remains a challenge. Some of lncRNAs were exhibited cancer type specificity and related to malignant transformation.^[13, 14, 15]^ Many genomic mutations were discovered not in protein encoding region, while they were inside lncRNAs transcribed location.^[15,16]^ By interacting with DNA, RNA and proteins, lncRNAs take part in gene regulatory networks, which have important implications for cancer therapeutic strategy. However, we are far from incorporating lncRNAs into the clinic, which is impeded by the limitation of fully understanding their mechanisms.^[4]^ It is a big challenge to identify the full-length sequences and structural elements to develop novel, rational, tailored therapeutic strategies.

Here, we identified a novel lncRNA, Lnc5q21.2, and dug its role in CRC. Lnc5q21.2 promotes CRC growth by activating Wnt signaling *via* interacting with homeobox A10 (HOXA10) and its highly expression increased the sensitivity of ATR inhibitor.

## Materials and methods

### Cancer cell lines and tissue samples

All CRC cells were established from primary CRC. Normal colorectal mucosa from noncancerous patients (52), adenomas (146) and matched CRC and adjacent tissue samples (285) were obtained from the Chinese PLA General Hospital, under the guidelines approved by the institutional review board at the Chinese PLA General Hospital (NO. 20090701-015). Informed consent was obtained from all patients.

### Identification of Lnc5q21.2 with northern blot and obtaining its full-length sequence by RACE assay

Biotin RNA labeling mix (Roche, Switzerland, Cat#11685597910) and T7 RNA polymerase (Life, USA, Cat#AM2718) were utilized to synthesize Lnc5q21.2 probes for northern blot. The full-length sequence was obtained by 5’ and 3’ rapid amplification of cloned cDNA ends (RACE) assay according to the instruction of the FirstChoice RLM-RACE Kit (Ambion, USA, AM1700). The primer sequences are listed in Supplementary Table S1.

### RT-PCR and Lnc5q21.2 stably expressed and deleted cells establishment

RNA preparation and semi-quantitative reverse transcription polymerase chain reaction (RT-PCR) were followed previously.^[17]^ SYBR Green was applied for Real-time RT-PCR. GAPDH was used as an internal reference to calculate 2^−ΔCt^, representing the relative expression level of lncRNAs.^[18]^ Primer sequences for Lnc5q21.2 and GAPDH are listed in Supplementary Table S1.

The human full-length cDNA of Lnc5q21.2 was cloned into the pLenti6-GFP vector for stably expressed cells. The primer sequences were listed in Supplementary Table S1. The single guide RNA (sgRNA) sequences for targeting Lnc5q21.2 were designed by zlab (https://www.zlab.bio/resources). Monoclonal Lnc5q21.2 deleted cells were selected by limited dilution and verified by PCR. The primer sequences were listed in Supplementary Table S2.

Lnc5q21.2 was cloned into pcDNA3.1+ vectors for transient expression. The full-length and three possible distinct coding sequences were constructed. The possible starting sites (ATG) are as follow 5’-ATGATATTGG…Flag-3’, 5’-ATGTATTGG… Flag-3’ and 5’-ATGATTGG… Flag-3’. TMEM176A-pcDNA3.1-3×Flag plasmid was used as control.^[19]^

### Fluorescence in situ hybridization

To clarify the location of Lnc5q21.2 in cells, fluorescence *in situ* hybridization assay (FISH) was employed. Probes for Lnc5q21.2 detection were designed according to LNA probe software (https://sg.idtdna.com/pages), which were labeled with DIG following the manufacturer’s instruction. Lnc5q21.2 highly expressed HCT116 cells were plated on sterile glass coverslips and incubated for 24 h. Cells were washed with PBS and fixed with 4% formaldehyde for 15 min at room temperature. Then, the cells were permeabilized in PBS containing 0.5% Triton X-100 on ice for 5 min, and washed for 3 times using PBS. Labeled Lnc5q21.2 probes were applied for FISH following the protocols of TSA ™ plus fluorescence systems (PerkinElmer, USA, Cat# NEL741). A Leica TCS SP2 confocal laser microscopy was utilized to observe the location of labeled Lnc5q21.2 probes in cells. The labeled probe sequences for Lnc5q21.2 were listed in Supplementary Table S3.

### RNA pull down

To gain the interacting proteins of Lnc5q21.2, six distinct parts of Lnc5q21.2 were labeled by biotin to serve as detection probes. The labeled lacZ mRNA probes were served as negative control (BGI, China). The sequences of these probes are listed in Supplementary Table S3. Cell lysate prepared from HCT116 cells was applied for RNA pull down.^[20]^

By using RNA pull down, the interacting sequence of Lnc5q21.2 to related protein was obtained by truncating Lnc5q21.2. Different parts of Lnc5q21.2 fragments were labeled by biotin using *in vitro* transcription assay, including Lnc5q21.2F1-200, Lnc5q21.2F1-400, Lnc5q21.2F1-668, Lnc5q21.2F200-668 and Lnc5q21.2F400-668.

### RNA immunoprecipitation and chromatin immunoprecipitation

To validate the interaction of proteins to Lnc5q21.2, RNA immunoprecipitation (RIP) approach was employed by using antibodies against proteins gained from RNA pull down.^[20]^ The antibodies were listed in Supplementary Table S4.

Chromatin immunoprecipitation (ChIP) was performed according to the instruction of EpiTect ChIP One Day Kit (Qiagen, Germany, Cat# 334471). The primers sequences for promoter region detection were listed in Supplementary Table S1.

### Chromatin isolation by RNA purification assay

To find more clues for Lnc5q21.2 interacting with empty spiracles homeobox 2 (EMX2), Chromatin isolation by RNA purification (ChIRP) assay was utilized.^[21]^ Using labeled Lnc5q21.2 probes, the promoter region sequence of EMX2 gene was detected from DNA extracted from cross-linked HCT116 cells lysates.

### Additional materials & methods

Part of material & methods were described in the supplementary data, including MTT, colony formation, flow cytometry, transwell assays and xenograft mouse model, nucleus and cytoplasm isolation, luciferase reporter assay and siRNA knockdown.

### Statistical analysis

SPSS 23.0 software (IBM, USA) for χ^2^, Fisher’s exact and Student’s *t*-test were used for statistical analysis. Kaplan-Meier plots and the log-rank test were used to estimate overall survival (OS). The five-year OS was assessed by univariate and multivariate Cox proportional hazards regression models. *P* < 0.05 was determined as significance difference.

## Results

### Lnc5q21.2 was highly expressed in CRC

**Figure 1:**
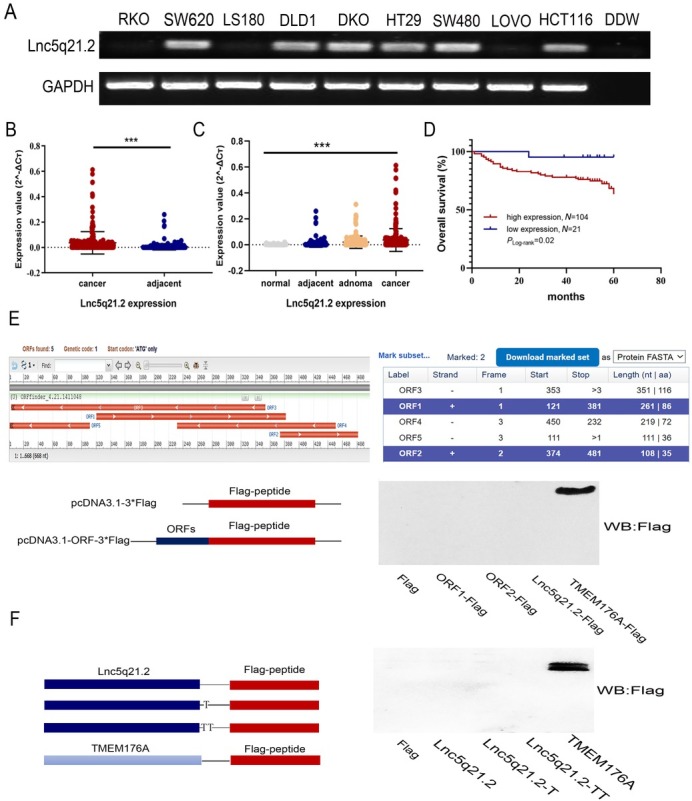
The expression of Lnc5q21.2 in CRC. A: Semi-quantitative RT-PCR shows the expression of Lnc5q21.2 in CRC cells. DDW: double distilled water; GAPDH, internal control. B: The expression of Lnc5q21.2 in normal colorectal mucosa, adenoma, CRC and adjacent tissue samples. normal: normal colorectal mucosa; adjacent: adjacent tissue samples; adenoma: colonic adenoma samples; cancer: CRC samples. C: The expression of Lnc5q21.2 in CRC and adjacent tissue samples.D: The association of Lnc5q21.2 and 5-year OS of CRC. E: The potential predicted ORFs was presented by ORF Finder. ORF1, 121-381bp; ORF2, 374-481bp, ORF3, 353-3bp, ORF4, 450-232bp, ORF5, 111-1bp; +, forward; -, reverse. TMEM176A with Flag tag severs as a positive control. F: Full-length Lnc5q21.2 was cloned into the eukaryotic expression vector pcDNA3.1 with three possible coding patterns. TMEM176A with Flag tag severs as a positive control. ^***^P < 0.001. CRC, colorectal cancer; RT-PCT, reverse transcription polymerase chain reaction; OS, overall survival.

One of the lncRNA fragments was isolated differentially expressed from a small number of cancers and matched adjacent tissue samples by high-throughput assay (https://www.ncbi.nlm.nih.gov/geo/query/acc.cgi?acc=GSE70880). The original length of this fragment is 273 nt. It is located on human Chromosome 5q21.2 and named as Lnc5q21.2 according to the genomic location. To deep understand Lnc5q21.2 in CRC, the expression was detected in 9 CRC cell lines first. Lnc5q21.2 was highly expressed in HCT116, SW480, SW620, DLD1, HT29 and DKO cells, and unexpressed in RKO, LOVO and LS180 cells (Figure 1A). Then, the expression was examined in tissue samples. The expression level of Lnc5q21.2 was increased significantly in CRC tissue compared to adjacent tissue samples (*P* < 0.001, Figure 1B), with a progression tendency from noncancerous colorectal mucosa, adjacent tissue, adenoma to CRC samples (*P* < 0.001, Figure 1C). Increased level of Lnc5q21.2 was associated with alcohol (*P* < 0.05, Table 1), while no association with age, gender, tumor size, TNM stage, lymph node metastasis, smoking or family tumor history (all *P* > 0.05, Table 1).

**Table 1:** The association of Lnc5q21.2 expression and clinical factors in human CRC

Clinical parameter	NO. 285	High expression *n* = 236	Reduced/Loss expression *n* = 49		*P* value^*^
Gender					
Male	179	154	25		0.061
Female	106	82	24		
Age (years)					
≤50	48	41	7		0.599
>50	237	195	42		
Differentiation					
Moderately/Well	214	182	32		0.082
Poorly	71	54	17		
Tumor stage					
I/II	170	143	27		0.476
III/IV	115	93	22		
Metastasis					
Positive	107	83	24		0.069
Negative	178	153	25		
Tumor size					
≤4 cm	119	100	19		0.642
>4 cm	166	136	30		
Smoking					
No	197	159	38		0.161
Yes	88	77	11		
Drinking					
No	234	188	46		0.018*
Yes	51	48	3		
Inherited					
No	275	228	47		0.811
Yes	10	8	2		

*P values are obtained from χ^2^ test, significant difference, *P* < 0.05. CRC, colorectal cancer.

For 125 cases of available CRC with survival data, a Cox multivariable proportional hazards and Kaplan-Meier model were applied to analyze the association of Lnc5q21.2 expression and survival time. Lnc5q21.2 expression is significantly associated with poor 5-year OS (*P* < 0.05, Figure 1D) and is an independent prognostic factor (*P* < 0.05, Table 2). These results demonstrated that Lnc5q21.2 is a poor prognostic marker.

**Table 2:** Univariate and multivariate analysis of Lnc5q21.2 expression with overall survival in CRC patients

Clinical parameter	Univariate analysis		Multivariate analysis	
	HR (95%CI)	*P* value	HR (95%CI)	*P* value
Gender (male *vs*. female)	0.580 (0.283,1.189)	0.137		
Age (>50 *vs*. ≤50 years)	0.651 (0.266,1.597)	0.349		
Tumor size (>4 *vs*. ≤4 cm)	0.899 (0.439,1.840)	0.771		
Differentiation (low *vs*. high or middle differentiation)	2.591 (1.257,5.342)	0.010^*^	2.598 (1.234,5.470)	0.012^*^
TNM stage (III/IV *vs*. I/II)	6.609 (2.694,16.213)	0.000^***^	7.358 (1.486,36.425)	0.014^*^
Lymph node metastasis (positive *vs*. negative)	4.889 (2.169,11.020)	0.000^***^	0.881 (0.203,3.812)	0.865
Lnc5q21.2 (high expression *vs*. low expression)	7.501 (1.017,55.346)	0.048^*^	10.388 (1.396,77.319)	0.022^*^
Smoking (yes *vs*. no)	0.842 (0.385,1.840)	0.666		
Drinking (yes *vs*. no)	0.511 (0.155,1.685)	0.270		
Inherited (yes *vs*. no)	0.492 (0.117,2.068)	0.333		

HR: hazard ratio; CRC, colorectal cancer. ^*^P < 0.05; ^***^P < 0.001.

The association of Lnc5q21.2 and microsatellite instability (MSI) was also evaluated by detecting mismatch repair genes (MMR) expression with immunohistochemistry, including MLH1, MSH2, MSH6 and PMS2. Deficiency of MMR (dMMR) was observed in 9.6% (12/125) of cases. KRAS mutation was found in 8% (10/125) and BRAF was mutated in 4.8% (6/125) of patients. No association was found between Lnc5q21.2 expression and dMMR, KRAS or BRAF mutation (all *P* > 0.05, Supplementary Table S5).

### Lnc5q21.2 transcript is a new long non-coding RNA

First, the transcript Lnc5q21.2 was evaluated by Northern Blot, and then the full-length sequence was obtained by 5’ and 3’ RACE technique for 668nt (Supplementary Table S6), validating that Lnc5q21.2 is a novel long intergenic non-coding RNA with a poly (A) tail (Supplementary Figure S1A–C). Analyzing by nuclear- cytoplasmic separation and FISH assays, Lnc5q21.2 was displayed mainly located in the nucleus of HCT116 cells (Supplementary Figure S1D and E).

The potential coding ability for protein or peptic coding was estimated using Coding Potential Assessment Tool (CPAT) (http://lilab.research.bcm.edu/cpat/index.php), Coding Potential Calculator (CPC, https://cpc.gao-lab.org/),^[22]^ LGC (https://ngdc.cncb.ac.cn/lgc/),^[23]^ PLEK (https://sourceforge.net/projects/plek/)^[24]^ and ORF Finder (https://www.ncbi.nlm.nih.gov/orffinder/). Lnc5q21.2 was predicted possibly encoding two peptides according to ORF Finder software. No protein or peptide coding ability was found by other databases (Figure 1E and Supplementary Table S7). To further evaluate the possibility of peptide coding ability of Lnc5q21.2, the full-length sequence and two predicted peptide ORFs were cloned into pcDNA3.1-3xFlag expression vector. TMEM176A, encoding a short protein sequence, was used as the control. Supposing exists three possible translational start sites at the 5’ of Lnc5q21.2, various expressing constructs were built by selecting three different start site. These expression constructs include the full length Lnc5q21.2. sequence and the potential nucleotide sequences for predicting to coding peptides. No fusion protein was found by expressing these constructs in RKO cells (Figure 1E–F), excluding the protein/peptide coding ability of Lnc5q21.2.

### Up-regulating Lnc5q21.2 by 6mA modification

The promoters of lncRNAs were reported almost as conservative as protein-coding genes and their expression regulation is also similar.^[25]^ RNA transcription may be also regulated by N^6^-methyladenine (6mA) DNA modification.^[26,27]^ While no CpG islands were discovered by analyzing the sequence around transcription start site (TSS), excluding promoter region methylation to regulate Lnc5q21.2 expression. Then, ChIP assay was employed to assess the possibility of histone modulation of Lnc5q21.2 expression. No apparent difference was observed for activating marker (H3K4me3) and inhibiting marker (H3K9me2) in Lnc5q21.2 highly expressed HCT116 and unexpressed RKO cells, indicating that Lnc5q21.2 was not regulated by histone modification (Supplementary Figure S2A).

DNA 6mA is related to transcriptional activation, and [G/C] AGG [C/T] is the most prevalent motif for 6mA modification.^[28]^ Existing “AGG” sequence around the TSS of Lnc5q21.2 hints the possibility of the expression regulation by 6mA modification. By analyzing with ChIP assay, DNA 6mA was found in Lnc5q21.2 expressed HCT116 cells, while no 6mA was detected in Lnc5q21.2 unexpressed RKO cells, indicating 6mA regulation of its expression (Supplementary Figure S2B). DNA 6mA modification was mediated by methyltransferase N6AMT1.^[28]^ Thereafter, siRNA was employed to knock down N6AMT1. DNA 6mA level was reduced after knockdown of N6AMT1 in HCT116 cells (Supplementary Figure S2C). The result further demonstrated that Lnc5q21.2 expression is regulated by 6mA modification.

### Lnc5q21.2 promotes CRC growth

**Figure 2:**
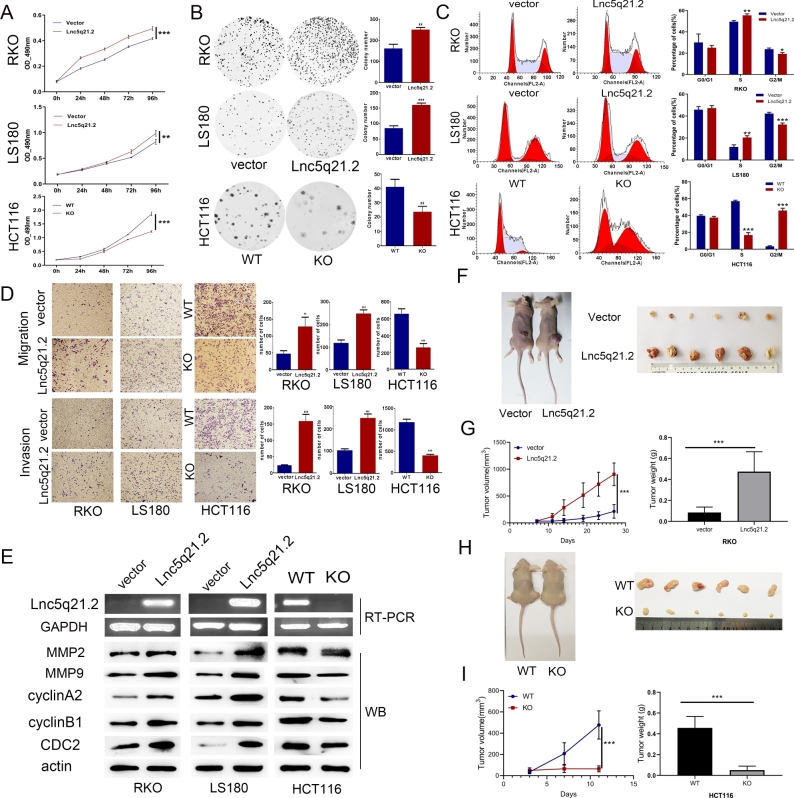
The role of Lnc5q21.2 in CRC cell growth. A: The OD value in Lnc5q21.2 expressed and unexpressed CRC cells. Each experiment was repeated for three times and OD value was shown as mean ± SD. B: Colony formation assay shows the efficiency of Lnc5q21.2 on CRC cells. Each experiment was repeated three times. C: Cell cycle distribution in Lnc5q21.2 unexpressed and expressed CRC cells. Each experiment was repeated three times. D: Transwell assay shows the role of Lnc5q21.2 in cell migration and invasion. Each experiment was repeated three times. E: Western blots show the levels of cell cycle, invasion and migration related molecules in Lnc5q21.2 expressed and unexpressed CRC cells. F: Represents results for Lnc5q21.2 unexpressed and re-expressed RKO cell xenografts. G: Tumor growth curves and tumor weight of Lnc5q21.2 unexpressed and overexpressing RKO cells. H: Represents results for Lnc5q21.2 highly expressed and knockout HCT116 cell xenografts. I: Tumor growth curves and tumor weight of Lnc5q21.2 highly expressed and knockout HCT116 cell. ^*^P < 0.05, ^**^P < 0.01, ^***^P < 0.001. CRC, colorectal cancer; WT, wild type; KO, knockout.

LncRNAs may play multifaceted function depending on their sequences and structure.^[25,29]^ To understand the role of Lnc5q21.2 in CRC, the full-length sequence was cloned into Lentiviral vectors to obtain Lnc5q21.2 stably expressed RKO and LS180 cells. Lnc5q21.2 was observed to promote CRC cell proliferation and migration, increase S phase and reduce G2/M phase cells. Above results were validated by knockout of Lnc5q21.2 in HCT116 cells (Figure 2A-E and Supplementary Figure S3), suggesting the oncogenic role of Lnc5q21.2 in CRC cells.

To further estimate the effect of Lnc5q21.2 on CRC, RKO cell xenograft mice were utilized. The tumor volume was 176.3 ± 126.4 mm^3^ *vs*. 957.8 ± 209.9 mm^3^ in Lnc5q21.2 silenced and overexpressed xenografts, increasing the tumor volume by Lnc5q21.2 (*P* < 0.001, Figure 2F and 2G). The tumor weight was 0.09 ± 0.05 g *vs*. 0.47 ± 0.19 g in Lnc5q21.2 silenced and overexpressed xenografts, increasing the tumor weight by Lnc5q21.2 (*P* < 0.001, Figure 2G). The promoting role of Lnc5q21.2 in CRC growth was verified by CRISPR/Cas9 knockout technique. The tumor volume was 477.9 ± 132.7 mm^3^ *vs*. 64.4 ± 28.1 mm^3^ in Lnc5q21.2 highly expressed and deleted HCT116 cell xenografts. The tumor volume was decreased by depletion of Lnc5q21.2 (*P* < 0.001, Figure 2H and 2I). The tumor weight was 0.457 ± 0.110 g *vs*. 0.051 ± 0.039 g in Lnc5q21.2 highly expressed and deleted HCT116 cell xenografts. Reduced tumor weight was observed in Lnc5q21.2 depleted HCT116 cell xenografts (*P* < 0.001, Figure 2I). Lnc5q21.2 promotes CRC cell xenograft growth in mice.

### Lnc5q21.2 was discovered to interact with HOXA10

**Figure 3:**
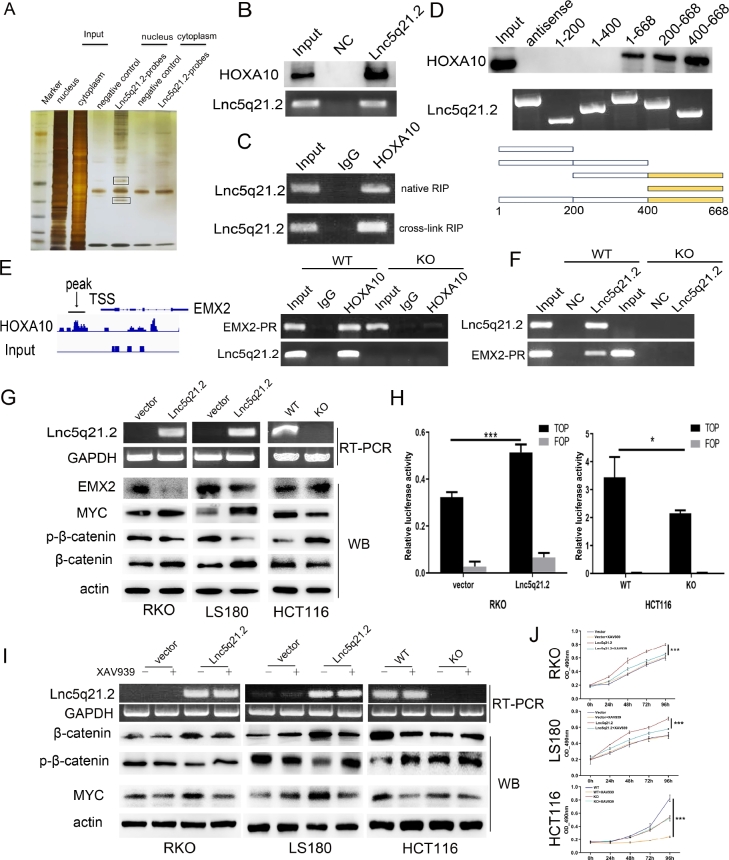
Lnc5q21.2 interacting with HOXA10 and promotes the Wnt signaling pathway. A: RNA pull-down assay shows the interacting of Lnc5q21.2 with proteins. The SDS-PAGE was stained with silver. The labeled bands were resected for mass spectrometry. B: Western blot validation for the specificity biding of Lnc5q21.2 and HOXA10 by HOXA10 antibody after RNA pull-down. C: Lnc5q21.2 enrichment in native and cross-linked RIP. D: One region in the 3’ end of Lnc5q21.2 is necessary to associate with HOXA10. Different Lnc5q21.2 fragments were used for RNA pull-down assay. The combination of Lnc5q21.2 fragment was revealed by western blot using HOXA10 antibody. E: ChIP-seq and ChIP-PCR results indicate HOXA10 binding to the promoter region of EMX2. F: ChIRP assay shows the promoter region of EMX2 existed in the complex obtained by Lnc5q21.2 probes, suggesting the interaction. G: Western blots show the effect of Lnc5q21.2 on the expression levels of EMX2, myc, β-catenin and p-β-catenin in RKO, LS180, and HCT116 cells. Semi-RT-PCR demonstrated that the expression of Lnc5q21.2 and GAPDH in CRC cells. vector, control vector; Lnc5q21.2, Lnc5q21.2 expression vector; actin, internal control; WT, Lnc5q21.2 high expression control; KO, Lnc5q21.2 expression knockout; GAPDH, internal control. H: Results of TCF/LEF luciferase reporter assay. I: Western blot shows the levels of myc, β-catenin and p-β-catenin in Lnc5q21.2 expressed and unexpressed CRC cells before and after XAV939 treatment. actin: internal control. J: Growth curves represent cell viability evaluated by MTT assay in CRC cells with or without XAV939(10 μmol/L) treatment. Each experiment was repeated for three times and OD value was shown as mean ± SD. ^*^P < 0.05, ^***^P < 0.001. HOXA10, meobox A 10; ChIP, chromatin immunoprecipitation; CRC, colorectal cancer; PCR, polymerase chain reaction.

To further understand the mechanism of Lnc5q21.2 in CRC, RNA pulldown and mass spectrometry techniques were employed. Two extra bands were obtained from the nuclear lysates of Lnc5q21.2 highly expressed HCT116 cells and analyzed by mass spectrometry (Figure 3A). Among Lnc5q21.2 binding proteins, HOXA10 was discovered and validated by RNA pulldown and western blot techniques (Figure 3B). Lnc5q21.2 binding to HOXA10 was reproduced by RIP assay using HOXA10 antibody in crosslinking treated and untreated HCT116 cell lysates (Figure 3C).

The evidence of Lnc5q21.2 binding to HOXA10 was further acquired by leveraging Lnc5q21.2 truncating fragments. The binding sequence of Lnc5q21.2 to HOXA10 was the fragment F400-668 (Figure 3D).

### Lnc5q21.2 activates Wnt signaling by HOXA10 in CRC cells

To deeply understand the mechanism of Lnc5q21.2 interacting with HOXA10, the ChIP-sequence (ChIP-seq) and ChIP-PCR were borrowed. By using HOXA10 antibody, the combination of HOXA10 to the promoter region of EMX2 was discovered by ChIP-seq and validated by ChIP-PCR. At the same time, the sequence of Lnc5q21.2 was also discovered in the binding complex, suggesting that HOXA10 binds to both Lnc5q21.2 and the promoter region of EMX2 (Figure 3E). Labeled Lnc5q21.2 probes were used for ChIRP technique. The promoter region of EMX2 sequence was detected in the complexes of Lnc5q21.2 highly expressed HCT116 cells, while it did not emerge in Lnc5q21.2 unexpressed cells, further suggesting that Lnc5q21.2 binds to HOXA10 and EMX2 promoter region simultaneously (Figure 3F). The role of Lnc5q21.2 involves in EMX2 expression regulation was further validated by detecting the levels of EMX2 in Lnc5q21.2 highly expressed and unexpressed CRC cells (Supplementary Figure S4).

EMX2 mutation causes developmental defects in *Drosophila melanogaster* and homozygous mutants produced by intercrossing of heterozygotes died soon after birth in mice.^[30]^ The transcription of EMX2 has been shown to be repressed by HOXA10 through binding its promoter region, supporting our above findings.^[31]^

Both HOXA10 and EMX2 have been displayed to regulate the Wnt signaling in cancers, independently.^[32,33]^ The regulation of EMX2 by HOXA10 was reported previously.^[31,34]^ Our above results demonstrated that HOXA10 binds to the promoter region of EMX2 gene. The regulation of Wnt signaling by HOXA10 was verified by siRNA knockdown technique. As shown in Supplementary Figure S5, reduced the total level of β-catenin and myc was displayed in HCT116 cells, while increased the level of p-β-catenin was observed after knockdown of HOXA10.

Thereafter, we explored the regulation role of Lnc5q21.2 in Wnt signaling. The key proteins of Wnt signaling were detected by western blot in CRC cells with or without expression of Lnc5q21.2. Increased the levels of β-catenin and myc were observed in Lnc5q21.2 re-expressed RKO and LS180 cells, while the levels of EMX2 and p-β-catenin were reduced. The levels of β-catenin and myc were reduced, and the levels of EMX2 and p-β-catenin were increased by knocking out Lnc5q21.2 in HCT116 cells (Figure 3G). Then, the role of Lnc5q21.2 in Wnt signaling was further determined by analyzing its effect on the activity of TCF/LEF transcription with dualluciferase reporter assay. Increased the activity was shown significantly in β-catenin and Lnc5q21.2 co-transfected RKO cells, while the activity was reduced by deletion of Lnc5q21.2 in HCT116 cells (Figure 3H).

For further verifying the role of Lnc5q21.2 in Wnt signaling, a Wnt signaling inhibitor (XAV939) was employed. CRC cells were divided into four groups, including empty vector, empty vector + XAV939, overexpression of Lnc5q21.2, overexpression of Lnc5q21.2 + XAV939 treatment. The impact of Lnc5q21.2 on Wnt signaling was evaluated by detecting β-catenin and MYC with western blot. The level of β-catenin and MYC was reduced by XAV939 in Lnc5q21.2 re-expressed RKO and LS180 cells. While no obvious changes were observed for β-catenin and MYC by deleting Lnc5q21.2 in HCT116 cells (Figure 3I). These results provide more evidence for Lnc5q21.2 taking part in Wnt signaling.

To further reveal Lnc5q21.2 promotion of CRC cell proliferation by activating Wnt signaling, MTT assay was employed. The OD values were 0.604 ± 0.014, 0.615 ± 0.047, 0.799 ± 0.017 and 0.654 ± 0.016 in untreated empty vector, XAV939 treated empty vector, untreated Lnc5q21.2 re-expressed and XAV939 treated Lnc5q21.2 re-expressed RKO cells, respectively. In LS180 cells, the OD values were 0.499 ± 0.040, 0.494 ± 0.009, 0.708 ± 0.019, and 0.579 ± 0.007 in untreated empty vector, XAV939 treated empty vector, untreated Lnc5q21.2 re-expressed and XAV939 treated Lnc5q21.2 re-expressed LS180 cells, respectively. No significant difference was displayed before and after treatment with XAV939 in Lncq5q21.2 unexpressed CRC cells (all *P* > 0.05, Figure 3J), while reduced the OD value was observed by XAV939 in Lncq5q21.2 re-expressing CRC cells (all *P* < 0.001, Figure 3J). These effects were verified by deleting Lnc5q21.2 in HCT116 cells. The OD values were 0.825 ± 0.051, 0.245 ± 0.010, 0.541 ± 0.017, and 0.523 ± 0.040 in Lnc5q21.2 highly expressed cells without treatment, XAV939 treatment, deletion of Lnc5q21.2, and deleting Lnc5q21.2 + XAV939, respectively. No significant changes were observed between Lnc5q21.2 deleting and Lnc5q21.2 deleting + XAV939 groups (*P* > 0.05). Nevertheless, the OD value was reduced significantly in the wild type HCT116 cells under the treatment of XAV939 (*P* < 0.001, Figure 3J). Above evidence further demonstrate that Lnc5q21.2 promotes CRC cell growth by activating Wnt signaling.

### Lnc5q21.2 promotes ATR activity by activating Wnt signaling in CRC cells

**Figure 4:**
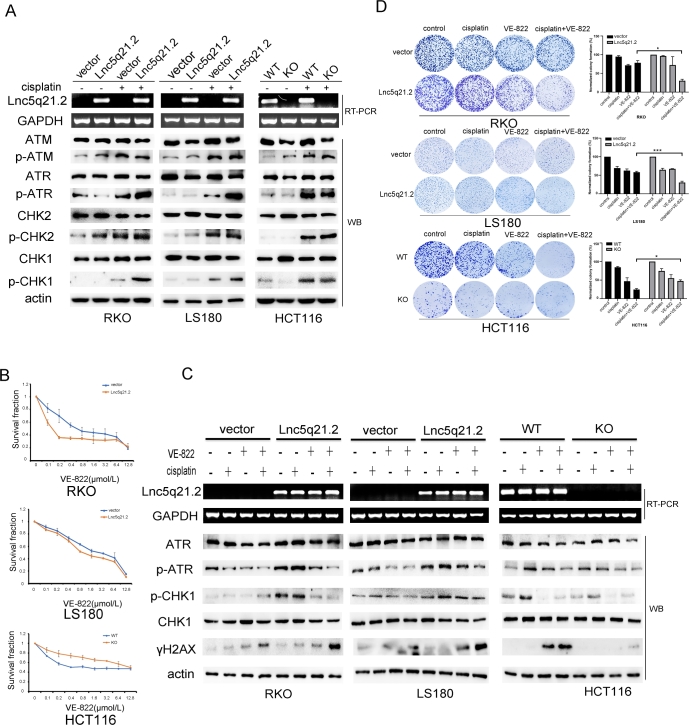
Lnc5q21.2 sensitives CRC cell to ATR inhibitor. A: Western blot shows the levels of ATM, ATR, p-ATM, p-ATR, CHK1, CHK2, p-CHK1 and p-CHK2 in Lnc5q21.2 expressed and unexpressed CRC cells before and after cisplatin treatment. Actin: internal control. B: The IC50 curve of VE-822 in CRC cells. C: Western blot shows the levels of ATR, p-ATR, CHK1, p-CHK1 and γH2AX in Lnc5q21.2 expressed and unexpressed CRC cells before and after cisplatin or VE-822 treatment. actin: internal control. D: Representative colony formation results to show the synthetic lethality between Lnc5q21.2 and ATR inhibitor. CRC, colorectal cancer. ^*^P < 0.05, ^***^P < 0.001.

It was noticed that HOXA10 is involved in homologous recombinant DNA repair (HR), and the Wnt signaling is also related to DDR.^[35,36]^ It is possible that Lnc5q21.2 involves in HR. The key regulators of HR were examined utilizing western blots. No apparent changes were observed for total levels of ATM, ATR, CHK2, CHK1, and the levels of p-ATM and p-CHK2, whilst the levels of p-ATR and p-CHK1 were increased by re-expressing Lnc5q21.2 in RKO and LS180 cells under the low dose cisplatin treatment, indicating that Lnc5q21.2 activated ATR signaling (Figure 4A). Similar results were obtained in HCT116 cells. The total levels of ATM, ATR, CHK2, CHK1, p-ATM and p-CHK2 were not changed before and after deletion of Lnc5q21.2, the levels of p-ATR and p-CHK1 were decreased after deletion of Lnc5q21.2 (Figure 4A).

The role of Lnc5q21.2 in ATR signaling was further verified using VE- 822, an ATR inhibitor. Under low dose cisplatin treatment, the IC50 value of VE-822 was 1.09 ± 0.16 *vs*. 0.13 ± 0.04 (*P* < 0.001), and 2.22 ± 0.42 *vs*. 1.37 ± 0.14 (*P* < 0.05) μmol/L in Lnc5q21.2 silenced and reexpressed RKO and LS180 cells, respectively. The IC50 was reduced significantly by forced re-expressing Lnc5q21.2 (Figure 4B). The IC50 value was 2.10 ± 0.49 *vs*. 13.30 ± 2.41 μmol/L in wild type and Lnc5q21.2 deleted HCT116 cells, increasing the value by depletion of Lnc5q21.2 (*P* < 0.01, Figure 4B).

By utilizing AZD0156, an ATM inhibitor, the role of Lnc5q21.2 in ATM signaling was further tested. The IC50 of AZD0156 was 17.47 ± 0.58 *vs*. 16.96 ± 0.61 and 13.45 ± 0.93 *vs*. 14.32 ± 0.13 μmol/L in Lnc5q21.2 unexpressed and re-expressed RKO and LS180 cells, independently. The IC50 was 14.46 ± 0.48 *vs*. 15.04 ± 1.66 μmol/L in wild type and Lnc5q21.2 deleted HCT116 cells. No significant effect was observed by AZD0156 with or without Lnc5q21.2 expression (all *P* > 0.05, Supplementary Figure S6). These results further reflect that Lnc5q21.2 may drive ATR signaling, without influencing ATM signaling.

The role of Lnc5q21.2 in ATR signaling was further tested by using VE822. With the treatment of cisplatin, the levels of γ-H2AX were increased and the levels of p-CHK1 and p-ATR were reduced by VE822 in Lnc5q21.2 re-expressed RKO and LS180 cells. Accordingly, the levels of γ-H2AX were increased and the levels of p-CHK1 and p-ATR were reduced in Lnc5q21.2 highly expressed HCT116 cells, indicating that Lnc5q21.2 promotes ATR signaling (Figure 4C).

The influence of Lnc5q21.2 in ATR signaling was evaluated by colony formation. Lnc5q21.2 unexpressed and expressed cells were treated with cisplatin, VE-822 and cisplatin+VE-822 and the colony formation efficiency was normalized by untreated empty vector group. Under these three different treatments, the colony formation efficiency was 94.97% ± 2.53% *vs*. 96.51% ± 1.36% (*P* > 0.05), 71.71% ± 2.50% *vs*. 72.43% ± 22.40% (*P* > 0.05) and 78.45% ± 5.88% *vs*. 30.40% ± 4.01% (*P* < 0.05) in RKO cells without and with expression of Lnc5q21.2. It was 69.47% ± 4.52% *vs*. 64.87% ± 3.99% (*P* > 0.05), 62.70% ± 4.31% *vs*. 67.30% ± 0.64% (*P* > 0.05) and 58.33% ± 2.46% *vs*. 30.39% ± 3.73% (*P* < 0.001) in LS180 cells before and after re-expression of Lnc5q21.2. The efficiency was 84.69% ± 2.14% *vs*. 74.77% ± 6.22% (*P* > 0.05), 47.10% ± 9.24% *vs*. 55.26% ± 9.35% (*P* > 0.05) and 24.65% ± 2.73% *vs*. 47.60% ± 3.08% (*P* < 0.05) before and after deleting Lnc5q21.2 in HCT116 cells (Figure 4D). The results demonstrated that the colony formation efficiency was reduced by Lnc5q21.2 under cisplatin+VE-822 treatment. No significant difference was observed with cisplatin or VE-822 single reagent treatment group, with or without Lnc5q21.2 expression.

**Figure 5:**
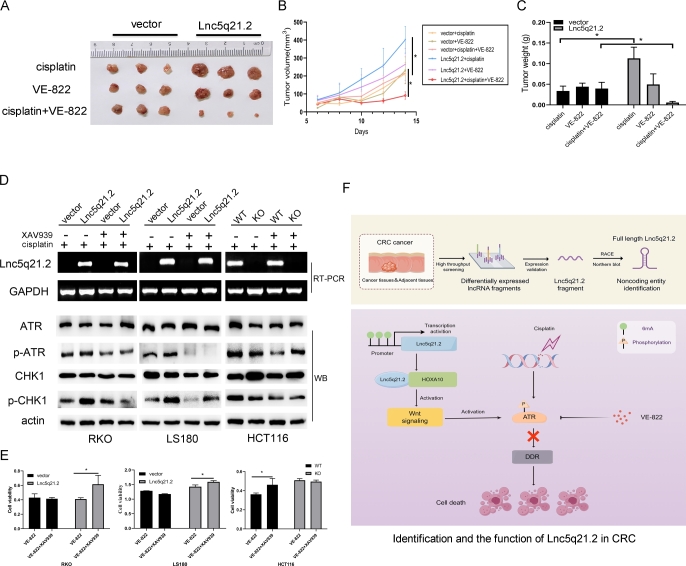
Lnc5q21.2 sensitives CRC cell to ATR inhibitor. A: Represents results for Lnc5q21.2 unexpressed and re-expressed RKO cell xenografts under various treatment. B: The growth curves of Lnc5q21.2 unexpressed and re-expressed RKO cell xenografts under various treatment. C: Tumor weight in Lnc5q21.2 unexpressed and re-expressed RKO cell xenografts under various treatment. D: The p-ATR and p-CHK1 was detected before and after XAV939 treatment under employing low dose cisplatin. XAV939, Wnt/β-catenin signaling inhibitor. E: Represent cell viability evaluated by MTT assay in CRC cells with or without XAV939 and VE-822 treatment. ^*^P < 0.05. F: Identification and the function of Lnc5q21.2 in CRC. CRC, colorectal cancer.

To further verify the sensitivity of Lnc5q21.2 to ATR inhibitor under cisplatin treatment, xenograft mouse models were used. Before and after re-expression of Lnc5q21.2, the tumor volumes were 213.38 ± 0.99 mm^3^ *vs*. 401.27 ± 73.92 mm^3^ (*P* < 0.05), 231.27 ± 75.07 mm^3^ *vs*. 265.23 ± 88.59 mm^3^ (*P* > 0.05), 217.26 ± 58.02 mm^3^ *vs*. 91.29 ± 20.68 mm^3^ (*P* < 0.05) in cisplatin, VE-822 and cisplatin plus VE-822 treatment, respectively. The tumor volume was reduced significantly in the combination of cisplatin and VE-822 treatment group by re-expressing Lnc5q21.2 (Figure 5A and 5B). In cisplatin, VE-822 and cisplatin plus VE-822 treatment groups, the tumor weights were 0.034 ± 0.012 g *vs*. 0.113 ± 0.027 g *(P* < 0.05), 0.044 ± 0.009 g *vs*. 0.050 ± 0.026 g (*P* > 0.05), 0.040 ± 0.015 g *vs*. 0.006 ± 0.003 g (*P* < 0.05) before and after re-expression of Lnc5q21.2, respectively. Reduced tumor weight was observed by cisplatin and VE- 822 treatment in Lnc5q21.2 re-expressed cell xenografts (Figure 5C). These results suggest that Lnc5q21.2 enforced the sensitivity of cisplatin treated CRC to VE- 822.

To get more clues for Lnc5q21.2 promoting ATR signaling by activating Wnt signaling, XAV939 was employed. Under low dose cisplatin treatment, the total levels of ATR and CHK1 were not changed with or without Lnc5q21.2 expression. While the levels of p-ATR and p-CHK1 were decreased by XAV939 in Lnc5q21.2 expressed CRC cells (Figure 5D).

To gain more evidence of Lnc5q21.2 activating ATR signaling through Wnt signaling, XAV939 and VE-822 were applied under low dose cisplatin treatment. The OD value was 0.431 ± 0.05 *vs*. 0.415 ± 0.02 (*P* > 0.05), and 0.411 ± 0.02 *vs*. 0.616 ± 0.12 (*P* < 0.05) for VE-822 or VE-822 plus XAV939 treatment groups, in Lnc5q21.2 unexpressed or over-expressed RKO cells, respectively. It was 1.29 ± 0.00 *vs*. 1.18 ± 0.01 (*P* > 0.05), and 1.43 ± 0.06 *vs*. 1.59 ± 0.04 (*P* < 0.05) for VE-822 or VE-822 plus XAV939 treatment groups, in Lnc5q21.2 unexpressed or over-expressed LS180 cells, respectively. In HCT116 cells, the OD value was 0.36 ± 0.01 *vs*. 0.47 ± 0.06 (*P* < 0.05) and 0.51 ± 0.02 *vs*. 0.49 ± 0.02 (*P* > 0.05) for VE-822 or VE-822 plus XAV939 treatment groups, before and after deletion of Lnc5q21.2, respectively. In Lnc5q21.2 unexpressed CRC cells, no significant difference was shown for ATR between Wnt plus ATR inhibitor groups (Figure 5E). It was significantly different in Lnc5q21.2 expressed cells for the treatment using ATR inhibitor and both Wnt and ATR inhibitors. The results were further validated by siRNA knockdown of β-catenin, and ICG-001, another Wnt signaling inhibitor (Supplementary Figure S7 and S8). These data suggest that Lnc5q21.2 promotes ATR signaling through Wnt signaling.

## Discussion

A recent study for the first time discovered that a lncRNA, which is overlapped with *cortex* gene, links to the evolution of a visible trait in animals, suggesting “the key regulator is RNA, not a protein”.^[5]^ In human genome, roughly 80% of diseases-related phenotype loci was identified to be associated to noncoding genome regions. Nonetheless, the linkage between phenotype-related loci and lncRNA-associated diseases almost remains uncharacterized.^[37]^ LncRNA has been recognized as important modulators in physiology and disease states, including carcinogenesis.^[4]^ Grasping cancer-related lncRNAs and unlocking their regulatory-circuits may garner diagnostic markers and innovate therapeutic strategies. In this study, we identified a novel lncRNA and its full-length sequence is 668 nt. The lncRNA was designated as Lnc5q21.2, owing to the genomic coding region locating in Chromosome 5q21.2. The expression of Lnc5q21.2 is regulated by 6mA in the promoter region. The location of molecules in subcellular may roughly reflect their role in cells. Lnc5q21.2 was detected mainly in the nucleus, implying to join the regulating action. The irregular expression of LncRNA hints the role in carcinogenesis and progression. The expression of Lnc5q21.2 was increased with a progression tendency from noncancerous colorectal mucosa to adjacent tissue, adenoma and CRC, reminding the possible oncogenic function. The high levels of Lnc5q21.2 is associated with alcohol and may serve as an independent poor 5-year OS marker. LncRNAs may serve as oncogenes to promote cancer growth, or tumor suppressors to inhibit tumor progression depending on distinct tumor types.^[38]^ Lnc5q21.2 promotes CRC cell proliferation, migration, invasion and cell cycle progression. It promotes tumor growth in CRC cell xenograft mice. These results provide some evidence for Lnc5q21.2 as an oncogenic molecule.

LncRNAs play their role by interacting with DNA, RNA and proteins.^[4]^ The interaction of Lnc5q21.2 with HOXA10 was revealed and verified by RNA pulldown, RIP and western blot techniques. The binding sequence was identified to be located at 400–668nt of Lnc5q21.2. Given that HOXA10 was involved in cell fate determining signaling, including DDR, Wnt, Sonic hedgehog and Notch signaling pathways.^[35,39,40,41]^ Next, we tried to find the target genes of HOXA10 through Lnc5q21.2. The combination of HOXA10, Lnc5q21.2 and the promoter region sequence of EMX2 was discovered by ChIP-seq and validated by ChIP-PCR and ChIRP assays using HOXA10 antibody and labeled nuclear probe from Lnc5q21.2. This interaction existed in Lnc5q21.2 highly expressed cells, without emerging in unexpressed cells. HOXA10 has been shown to regulate Wnt signaling *via* directly binding to the promoter region of EMX2.^[31,34,42]^ HOXA10 has been reported to be associated with chemo-resistance through regulating HR pathway, and Wnt signaling was verified to modulate cell fate determination and DDR.^[35,43,44]^ The role of Lnc5q21.2 in Wnt signaling was therefore explored. As expected, Lnc5q21.2 activated Wnt signaling through interacting with HOXA10 to modulate the expression of EMX2. To further understand the regulation network of Wnt signaling and HR, the mechanism of Lnc5q21.2 in HR was investigated. In CRC cells, Lnc5q21.2 enhanced ATR signaling by promoting Wnt signaling, while without influencing of ATM signaling.

With the deep insight into the mechanism of LncRNA, more attentions were paid for its application in cancer detection and therapeutics.^[45]^ Targeting DDR has been innovated cancer therapy.^[46,47]^ In CRC, Lnc5q21.2 increased the sensitivity of ATR inhibitor both *in vitro* and *in vivo*. In conclusions, Lnc5q21.2 is a novel long intergenic noncoding RNA. The expression of Lnc5q21.2 is regulated by 6mA modification. Highly expression of Lnc5q21.2 is associated with alcohol and serves as a potential independent poor prognostic marker. Lnc5q21.2 promotes ATR repair by activating Wnt signaling through interacting with HOXA10 *via* modulating EMX2 expression. Lnc5q21.2 plays an oncogenic role in CRC and serves as a sensitive marker for ATR inhibitor (Figure 5F).

## Supplementary Information

Supplementary materials are only available at the official site of the journal (www.intern-med.com).

## Supplementary Material

Supplementary Material Details
