# Supplementary material for: Lnc5q21.2, a novel long intergenic RNA, sensitizes colorectal cancer cells to ATR inhibitor by activating Wnt pathway
Source: J Transl Int Med. 2025 Oct 16;13(5):410–23. doi: 10.1515/jtim-2025-0040 (PMC12569581; doi:10.1515/jtim-2025-0040)
Supplement: Supplementary file 1 — Supplementary Material Details [file jtim-2025-0040_sm.pdf]

Supplementary materials

Lnc5q21.2, a novel long intergenic RNA, sensitizes colorectal cancer cells to ATR inhibitor by activating Wnt pathway

Meiying Zhang[1], Cheng Zhu[2,1], Aiai Gao[1], James G. Herman[3], François Fuks[4], Jianjun Luo[5], Xiaomo Su[1], Hengmi Cui[6], Runsheng Chen[5], Mingzhou Guo[1]

Authors' Affiliations:

1. Department of Gastroenterology & Hepatology, the First Medical Center, Chinese PLA General Hospital, #28 Fuxing Road, Beijing 100853, China
2. Medical College of NanKai University, Tianjin, 300071, China
3. The Hillman Cancer Center, University of Pittsburgh Cancer Institute, Pittsburgh, PA 15213, USA
4. Laboratory of Cancer Epigenetics, Free University of Brussels (U.L.B.), 808 route de Lennik, Brussels 1070, Belgium
5. Key Laboratory of RNA Biology, Institute of Biophysics, Chinese Academy of Sciences, Beijing 100101, China
6. Institute of Epigenetics and Epigenomics and College of Animal Science and Technology, Yangzhou University, 48 East Wenhui Road, Yangzhou, Jiangsu, 225009, China

***Correspondence:** Mingzhou Guo, PhD and MD, Department of Gastroenterology & Hepatology, Chinese PLA General Hospital, #28 Fuxing Road, Beijing 100853, China. Email: mzguo@hotmail.com.

Supplementary Text

Results

Lnc5q21.2 promotes CRC cell growth

The cell viability was evaluated by MTT assay. The OD values were 0.417 ± 0.008 *vs*. 0.495 ± 0.015 ($p < 0.001$) and 0.822 ± 0.050 *vs*. 0.986 ± 0.056 ($p < 0.01$) before and after re-expression of Lnc5q21.2 in RKO and LS180 cells, respectively (Fig. 2A). The OD value is increased significantly after re-expression of Lnc5q21.2 in CRC cells. The OD values were 1.863 ± 0.067 *vs*. 1.226 ± 0.028 before and after knockout of Lnc5q21.2 in HCT116 cells. The OD value is reduced significantly after deletion of Lnc5q21.2 ($p < 0.001$, Fig. 2A). These results indicate that Lnc5q21.2 promotes CRC cell proliferation.

The clone number was 161.333 ± 20.123 *vs*. 250.667 ± 10.066 ($p < 0.01$) and 85.33 ± 7.57 *vs*. 160.33 ± 6.11 ($p < 0.01$) before and after re-expression of Lnc5q21.2 in RKO and LS180 cells, respectively (Fig. 2B). The clone number is increased after restoration of Lnc5q21.2 expression in RKO and LS180 cells. The clone number was 46.00 ± 7.94 *vs*. 23.33 ± 3.06 before and after knockout of Lnc5q21.2 in HCT116 cells. The clone number was reduced significantly after knockout of Lnc5q21.2 ($p < 0.001$, Fig. 2B).

Lnc5q21.2 promotes cell cycle progression in CRC cells

The distribution of cell phases was 28.86 ± 2.17 *vs*. 24.69 ± 4.01 in G0/G1 phase ($p > 0.05$), 46.73 ± 0.64 *vs*. 56.35 ± 2.92 in S phase ($p < 0.01$) and 24.42 ± 1.73 *vs*. 18.96 ± 2.39 in G2/M phase ($p < 0.05$) before and after restoration of Lnc5q21.2

expression in RKO cells. In LS180 cells, the distribution of cell phases was 45.82 ± 2.75 *vs*. 47.21 ± 2.35 in G0/G1 phase ($p > 0.05$), 11.93 ± 1.94 *vs*. 20.49 ± 1.64 in S phase ($p < 0.01$) and 42.24 ± 1.17 *vs*. 32.29 ± 1.41 in G2/M phase ($p < 0.001$) before and after restoration of Lnc5q21.2 (Fig. 2C). Before and after deletion of Lnc5q21.2 in HCT116 cells (Fig. 2C), the distribution of cell phases was 39.62 ± 1.22 *vs*. 37.45 ± 1.47 in G0/G1 phase ($p > 0.05$), 57.05 ± 1.04 *vs*.16.76 ± 2.98 in S phase ($p < 0.001$), 3.33 ± 0.68 *vs*. 45.80 ± 2.78 in G2/M phase ($p < 0.001$).

Lnc5q21.2 promotes CRC cell migration and invasion

The number of migratory cells was 47.67 ± 8.96 *vs*.128.67 ± 27.79 and 119.33 ± 13.32 *vs*. 250.00 ± 16.46 in RKO and LS180 cells before and after re-expression of Lnc5q21.2, respectively. The number of migration cell is increased significantly after re-expression of Lnc5q21.2 in these cells ($p < 0.01$, $p < 0.001$, Fig. 2D). The number of migratory cells was 657.67 ± 59.05 *vs*. 258.33 ± 45.71 before and after knockout of Lnc5q21.2 in HCT116 cells. The number of migration cell is reduced significantly by deletion of Lnc5q21.2 in HCT116 cells ($p < 0.001$, Fig. 2D).

The number of invasive cells was 23.333 ± 1.528 *vs*. 159 ± 20.881 and 103.33 ± 6.81 *vs*. 248.33 ± 18.15 before and after re-expression of Lnc5q21.2 in RKO and LS180 cells, respectively. The number of invasive cells was increased significantly after re-expression of Lnc5q21.2 in CRC cells (all $p < 0.001$, Fig. 2E). The number of invasion cells was 1180.00 ± 70.00 *vs*. 403.00 ± 26.63 before and after knockout of Lnc5q21.2 in HCT116 cells ($p < 0.001$, Fig. 2D).

Validation of Wnt-ATR crosstalk by siRNA knockdown of β-catenin and ICG-

001

To verify the promoting role of Lnc5q21.2 in ATR signaling through Wnt signaling, siRNA knockdown technique and ICG-001 (another Wnt signaling inhibitor) were applied. Under low dose cisplatin treatment, the total levels of ATR and CHK1 were not changed before and after knockdown of β-catenin, in Lnc5q21.2 expressed and unexpressed cells (Supplementary Fig. S7A). While the levels of p-ATR and p-CHK1 were decreased by knocking down β-catenin in Lnc5q21.2 expressed cells, hinting Lnc5q21.2 influences ATR/CHK1 signaling activity via Wnt signaling (Supplementary Fig. S7A and B). Similar results were obtained by treating these cells with ICG-001 (Supplementary Fig. S7C).

Supplementary material & methods

MTT, colony formation, flow cytometry, transwell assays and xenograft mouse model

MTT, colony formation, flow cytometry and transwell assays were followed previous procedures[1]. Four weeks old Balb/c nude mice were divided into two groups (6 mice for each). RKO cells were injected subcutaneously into the dorsal left side (4×10^6 cells in 0.15 ml phosphate-buffered saline). Seven days after implantation, tumor volume was measured each 4 days for 24 days. Wildtype and Lnc5q21.2 deleted HCT116 cells (6×10^6 cells) were injected into the dorsal left side of mice. Tumor volume was measured each 4 days, starting at 3 days for a total of 12 days. Tumor volume was calculated according to the formula: $V = L \times W^2/2$ (mm^3), where V represents volume, L represents biggest diameter (mm), and W represents smallest diameter (mm). The procedures were approved by the Animal Ethics Committee of the Chinese PLA General Hospital.

Lnc5q21.2 unexpressed and over-expressed RKO cell xenograft mice were administrated for cisplatin (2 mg/kg), VE-822 (30 mg/kg), cisplatin + VE-822. Cisplatin was injected intraperitoneally and VE-822 was administrated by oral gavage twice for each week.

Isolating the ingredient of cell nucleus/cytoplasm

Nucleus and cytoplasmic ingredients were isolated by utilizing nucleo-cytoplasmic separation assay, following the instructions of Nuclear/Cytosol Fractionation Kit (Beyotime, China, Cat#P0028). U6 and GAPDH were served as the nuclear and

cytoplasmic ingredients controls, respectively. Primers were listed in Table S1.

Dual-Luciferase reporter assay, siRNA knockdown technique, immunohistochemistry assay and mutation detection

Dual-Luciferase reporter assay was used to evaluate the role of Lnc5q21.2 in Wnt signaling[2,3].

The sequences of siRNA for targeting HOXA10, N6AMT1, β-catenin and scrambled control duplex were listed in Table S10. By evaluating the efficiency of these siRNAs, siRNA#2 was shown to be the most effective one for HOXA10, and both siRNA#1 and siRNA#2 were very effective for N6AMT1, and siRNA#2 was most effective one for β-catenin.

MLH1, MSH2, MSH6 and PMS2 were detected by immunohistochemistry followed previous procedures[4] (Table S4).

KRAS codons 12, 13 and 61 and BRAF codon 600 were amplified by PCR and sequenced according to previously reports[5,6]. The primer sequences are listed in Table S2.

References

1. Yang W, Guo C, Herman JG, Zhu C, Lv H, Su X, et al. Epigenetic silencing of JAM3 promotes esophageal cancer development by activating Wnt signaling. Clin Epigenetics 2022; 14: 164.
2. Yu Y, Yan W, Liu X, Jia Y, Cao B, Yu Y, et al. DACT2 is frequently methylated in human gastric cancer and methylation of DACT2 activated Wnt signaling. Am J Cancer Res 2014; 4: 710-24.
3. Dong Y, Cao B, Zhang M, Han W, Herman JG, Fuks F, et al. Epigenetic silencing of NKD2, a major component of Wnt signaling, promotes breast cancer growth. Oncotarget 2015; 6: 22126-38.
4. Du W, Gao A, Herman JG, Wang L, Zhang L, Jiao S, et al. Methylation of NRN1 is a novel synthetic lethal marker of PI3K-Akt-mTOR and ATR inhibitors in esophageal cancer. Cancer Sci 2021; 112: 2870-83.

5. Morandi L, de Biase D, Visani M, Cesari V, De Maglio G, Pizzolitto S, et al. Allele specific locked nucleic acid quantitative PCR (ASLNAqPCR): an accurate and cost-effective assay to diagnose and quantify KRAS and BRAF mutation. PLoS One 2012; 7: e36084.
6. He T, Zhang M, Zheng R, Zheng S, Linghu E, Herman JG, et al. Methylation of SLFN11 is a marker of poor prognosis and cisplatin resistance in colorectal cancer. Epigenomics 2017; 9: 849-62.

Supplementary figures

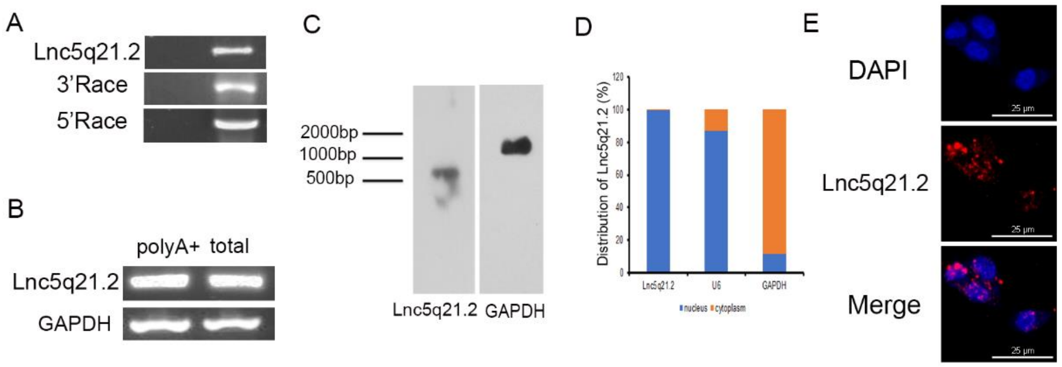

Supplementary Figure S1: The full length and location of Lnc5q21.2 in HCT116 cells.

A. The 5'RACE and 3'RACE of Lnc5q21.2. B. The poly (A) tail of Lnc5q21.2 was detected by olig d(T) RT-PCR. C. The length of Lnc5q21.2 was evaluated by northern blot. D&E.

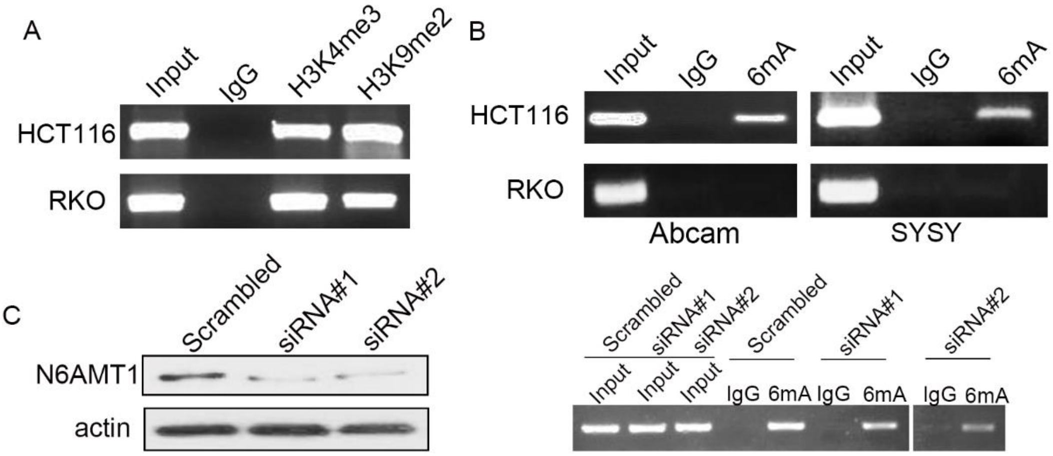

Supplementary Figure S2: In Lnc5q21.2 highly expressed CRC cells, 6mA modification was observed. A. histone modifications in the promoter region of Lnc5q21.2 detected by ChIP assay with H3K4me3 and H3K9me2 antibodies. B. The 6mA modification detected by ChIP assay using two bands of 6mA antibodies. C. The efficiency of siRNAs for N6AMT1. C. The 6mA modification was observed after knocking down N6AMT1.

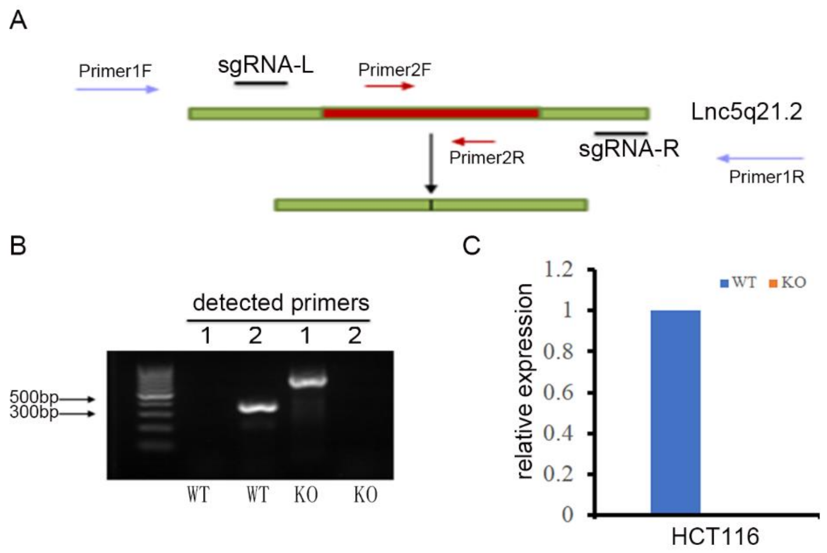

Supplementary Figure S3: The CRISPR/Cas9 knockout of Lnc5q21.2 in HCT116 cells.

A. The flow chart of CRISPR/Cas9 knockout assay. B. The validation of Lnc5q21.2 knockout in HCT116 cells. C. The expression of Lnc5q21.2 before and after knockout of Lnc5q21.2 in HCT116 cells.

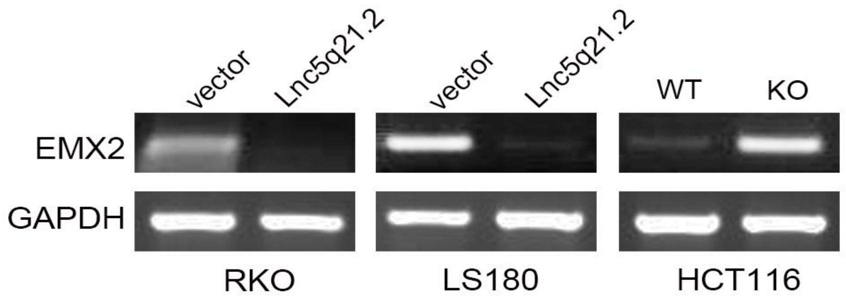

Supplementary Figure S4: The expression of EMX2 in Lnc5q21.2 unexpressed and expressed CRC cells.

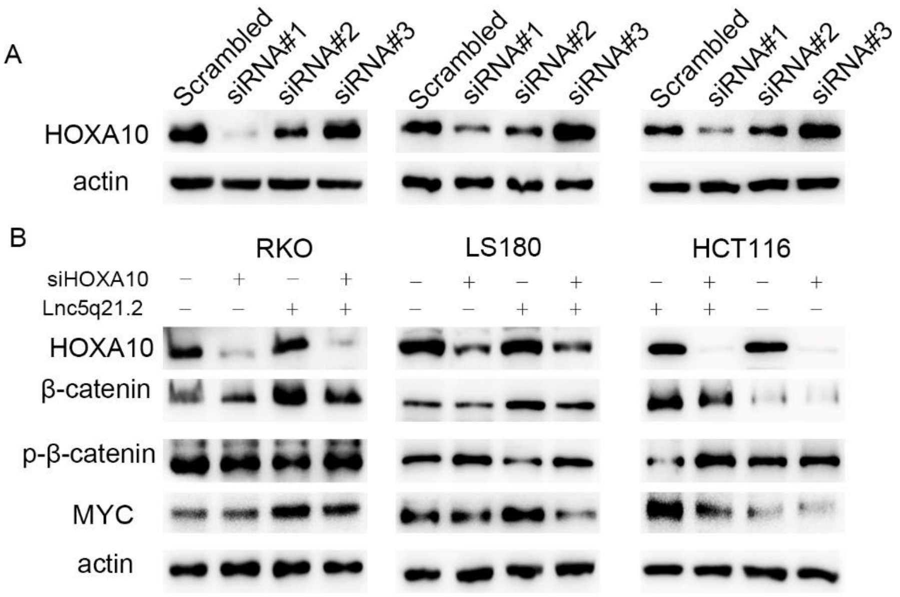

Supplementary Figure S5: The effect on Wnt/β-catenin signaling pathway after knockdown of HOXA10.

A. The efficiency of siRNAs for HOXA10. B. The levels of β-catenin, p-β-catenin and myc in CRC cells after knockdown of HOXA10.

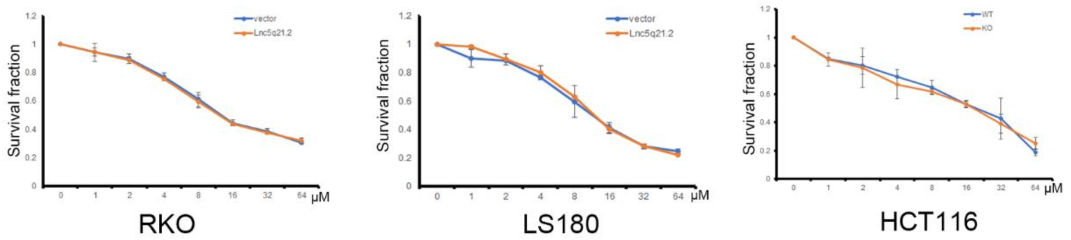

Supplementary Figure S6: The IC50 values of AZD0156 in Lnc5q21.2 expressed and unexpressed CRC cells.

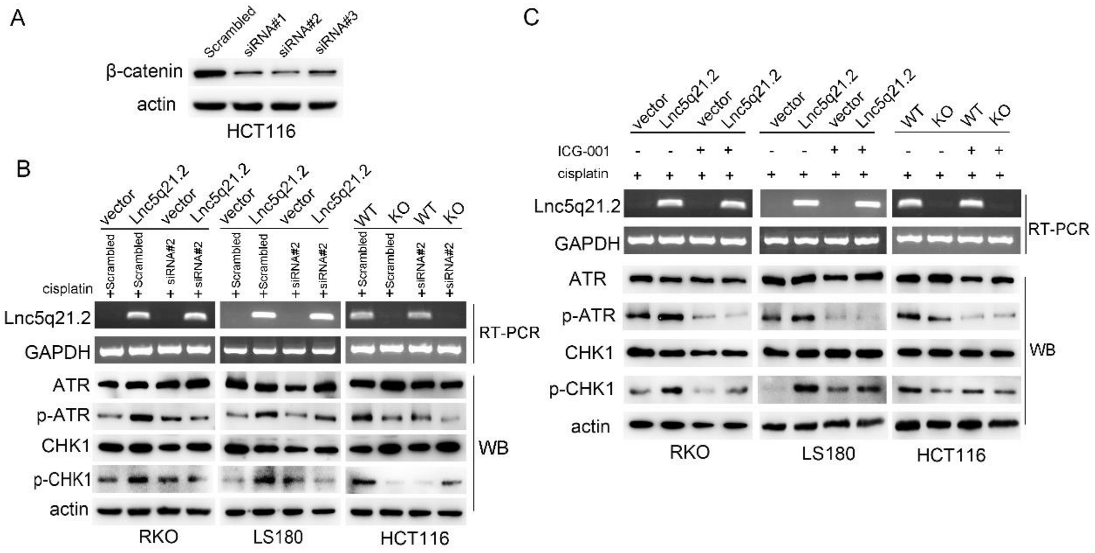

Supplementary Figure S7: SiRNA knockdown and ICG-001 treatment for validating Wnt-ATR crosstalk

A. The efficiency siRNAs for β-catenin. B. The levels of ATR, p-ATR, CHK1 and p-CHK1 in CRC cells before and after β-catenin knockdown under cisplatin treatment. C. The levels of ATR, p-ATR, CHK1 and p-CHK1 in CRC cells before and after ICG-001(10μmol/L) treatment in cisplatin treated cell models.

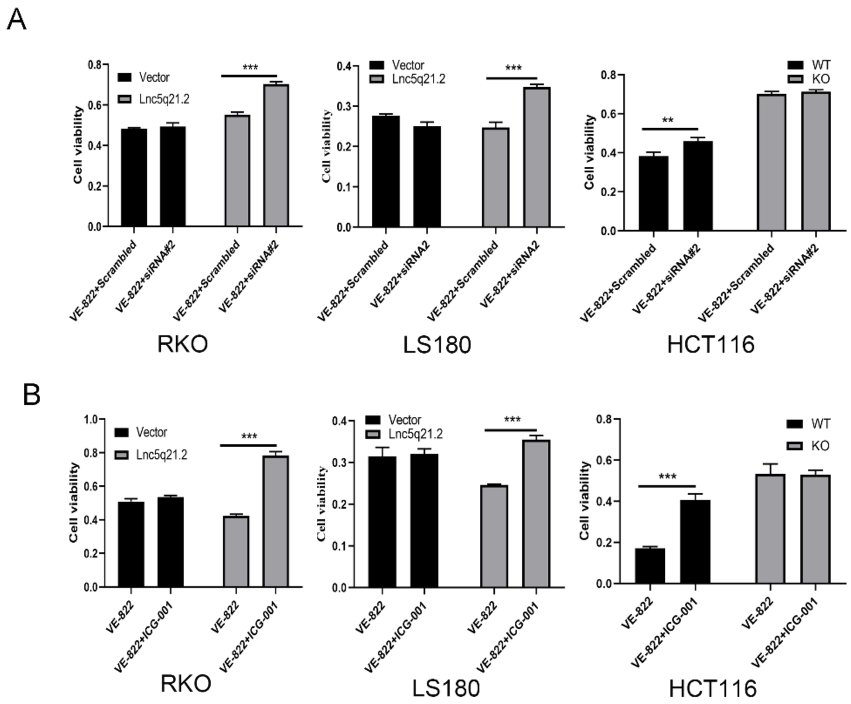

Supplementary Figure S8: VE-822 treatment and combined VE-822 with siRNA knockdown, or ICG-001 treatment to verify Wnt-ATR crosstalk

A. MTT assay to show the cell viability before and after β-catenin knockdown, under VE-822 treatment. $^{**}p < 0.01$, $^{***}p < 0.001$ B. The cell viability for VE-822 alone or combined ICG-001 treatment. $^{***}p < 0.001$

Supplementary Tables

Supplementary Table S1: Primers for PCR

Primer names	Sequences
Lnc5q.21.2 RT-PCR primer	5’-TGCCTGATATGCTCTCATTCTC-3’(F)<br>5’-TTTCTGCGCTCATTACAGACT-3’(R)
GAPDH RT-PCR primer	5’-GACCACAGTCCATGCCATCAC-3’(F)<br>5’-GTCCACCACCCTGTTGCTGTA-3’(R)
EMX2 RT-PCR primer	5’-ACTAGCCCCGAGAGTTTCCTTTTG-3’(F)<br>5’-CTCCAGCTTCTGCCTTTTGAACTTT-3’(R)
Lnc5q.21.2-5’RACE-primer	5’-CGCGGATCCGAACACTGCGTTTGCTGGCTTTGATG-3’(F)<br>5’-CCGCTCGAGGATAAATTTCATGCCATTTTCTGTTC-3’（R）
Lnc5q.21.2-3’RACE-primer	5’-GAGCTCTCACAGACCTTTTAATGC-3’(F)<br>5’-CGCGGATCCGAATTAATACGACTCACTATAGG-3’（R）
Lnc5q.21.2-full length primer	5’-CGCGGATCCATATTGGCCATAAT-3’(F)<br>5’-CCGCTCGAGTTTTTCAGTTCTAGTTAG-3’(R)
U6 RT-PCR primer	5’-CTCGCTTCGGCAGCACA-3’(F)<br>5’-AACGCTTCACGAATTTGCGT-3’(R)
EMX2 ChIP primer	5’-GGCTTCGGAGGAAGCTGTTT-3’(F)<br>5’-TCTCCTCTGCAAAAGGCGGG-3’(R)
Lnc5q.21.2 ChIP primer1	5’-TGAGTACATCTGATAAACAACAACA-3’(F)<br>5’-ACTTAAAGGTATGTTTACTGCTGTG-3’(R)
Lnc5q.21.2 ChIP primer2	5’-CACTCACTCAGTGACTCACCGA-3’(F)<br>5’-TTCCTAGGCTAGGAACCTGTGC-3’(R)
KRAS-2exon-primer	5’- AAGGTGAGTTTGTATTAAAAGGTACTGG-3’(F)<br>5’- TGGTCCTGCACCAGTAATATGC-3’(R)
KRAS-3exon- primer	5’- TCCAGACTGTGTTTCTCCCTTCTC-3’(F)<br>5’- AAAACTATAATTACTCCTTAATGTCAGCTT-3’(R)
BRAF-600E- primer	5’- TCATAATGCTTGCTCTGATAGGA-3’(F)<br>5’- CTTTCTAGTAACTCAGCAGC-3’(R)

Lnc5q.21.2 ChIP primer1: 6mA detection. Lnc5q.21.2 ChIP primer2: histone modification detected.

Supplementary Table S2: sgRNA and primers for CRISPR

Names	Sequences
Lnc5q.21.2-sgRNA-L	GCAGTGTACTGTAATGGCCT
Lnc5q.21.2-sgRNA-R	GTGGTCAAATAGTCAGGCTT
Lnc5q.21.2-primer1F	AGATGATCCTGACCCTGTG
Lnc5q.21.2-primer1R	AATGATGGGTTGTTGGTTA
Lnc5q.21.2-primer2F	AGTGTCACTCTGGGTTCGCT
Lnc5q.21.2-primer2R	AGTTCTAGTTAGAGCCTTTGTCCC

Supplementary Table S3: Probes

Probe names	Sequences
Lnc5q.21.2-probe1	AATCAGTTCTCCTGACAATCTTGAG
Lnc5q.21.2-probe2	TTTAAGATTGCCAAGCGAACCCAGA
Lnc5q.21.2-probe3	GCGCTCATTACAGACTGTTATGAGAATGGAT
Lnc5q.21.2-probe4	GTATCCCATTCCTGCCCTGTTACAA
Lnc5q.21.2-probe5	GTAATGAGAATGAGAGCATATCAGGC
Lnc5q.21.2-probe6	CTTTTTGGTTATGTTTGATGCATATTATGGCC
Lnc5q.21.2-LNA-probe	AGAATGAGAGCATATCAGGCA
Negative control probe1	GATCTTCCAGATAACTGCCG
Negative control probe2	TTAACGCCTCGAATCAGCAA
Northern primer	TTGGCCATAATATGCATCAAACAT(F)<br>TAATACGACTCACTATAGGGAAGATTGCCAAGCGAACCCA(R)

Supplementary Table S4: List of antibodies and inhibitors

Name	Manufacturer	Cat No.
Flag	ZENBIO	390002
MMP2	ZENBIO	HX15958
MMP9	Proteintech	10375-2-AP
MMP7	ZENBIO	820096
cyclinA2	Proteintech	18201-1-AP
cyclinB1	Proteintech	55004-I-AP
CDC2	Proteintech	19532-I-AP
actin	Proteintech	66009-1-Ig
HOXA10	Proteintech	26497-1-AP
EMX2	Abcam	ab174897
MYC	Proteintech	10828-1-AP
β-catenin	Proteintech	51067-2-AP
p-β-catenin	ZENBIO	310053
ATM	Hua XingBio	HX12561
p-ATM	Cell Signaling Technology	13050S
CHK2	ZEMBIO	R23921
p-CHK2	Cell Signaling Technology	2197S
ATR	Cell Signaling Technology	2790S
p-ATR	Cell Signaling Technology	2853S
CHK1	ZENBIO	380200
p-CHK1	Genetex	GTX100065
N6AMT1	Abcam	ab173804
m6A	Abcam	ab151230
m6A	Synaptic	202003
H3K4me3	Abclonal	A22226
H3K9me2	Abclonal	A2359
MLH1	ZSGB-BIO	ZM-0152
PMS2	ZSGB-BIO	ZA-0542
MSH2	ZSGB-BIO	ZA-0702
MSH6	ZSGB-BIO	ZA-0541
XAV-939	Selleck	S1180
VE-822	Selleck	S7102
AZD0156	Selleck	S8375
ICG-001	Selleck	S2662

Supplementary Table S5: KRAS, BRAF, MMR mutation and LncRNA5q21.2 expression status in CRC patients.

Clinical parameter	NO. 125	High expression n=104	Reduced/loss expression n=21	*P* value*
**KRAS status**				
Mutation	10	9	1	p=1.000
Wide-type	115	95	20	
**BRAF status**				
Mutation	6	6	0	p=0.588
Wide-type	119	98	21	
**MMR status**				
dMMR	12	9	3	p=0.423
MMR	113	95	18	

*P values are obtained from Fisher's exact test, significant difference, P<0.05

Supplementary Table S6: The full-length of Lnc5q21.2

Name	Sequence
**Lnc5q21.2**	AUAUUGGCCAUAAUAUGCAUCAAACAUAACCAAAA<br>AGAAAGAAAGAAGAGGGAGACAGGAGAGAGUAAAA<br>CAAAAUAAAAUAUAGAAAUGAUUUUUUUCAGUAAA<br>UAACAUUUAAUUGUGAUGCACAUGUUUUCAUGUAA<br>GUACUGUAUCACAGAAAAAAAUACUGUAAGCUUAA<br>AUACAAUCUCAAGAUUGUCAGGAGAACUGAUUAAU<br>UUUGCUCUCAUUCCCAUUUUCUCAACAAUCUUUGUC<br>UUCAGAGUGAAUAAAUACAGUGUCACUCUGGGUUC<br>GCUUGGCAAUCUUAAAAUUGUCACAAGAAUGGAUU<br>GGGUGCCGUUUCCUCUUCUUGUUACAAUCUUUGUC<br>AUGAGCUCUCACAGACCUUUUAAUGCCUGAUAUGC<br>UCUCAUUCUCAUUACAUAUUCUCAUUCCCAUAACAA<br>AGAUUAUAAUGAAAGAAGAAAAUGUCAUAAAAUUA<br>AUCCAUUCUCAUAACAGUCUGUAAUGAGCGCAGAA<br>AAUUGAGCAAAAUUAAUCCGUUCUCAUAACAAUCU<br>GAAGAUUGUAAUGAGAACAGAAAAUGGCAUGAAAU<br>UUAUCCUUCUUGUGACAAUCUUUAAAUUGUAACAG<br>GGCAGGAAUGGGAUACAAGAUUAACAAGGCCCUGG<br>GACAAAGGCUCUAACUAGAACUGAAAAAAAAAAAA<br>A

Supplementary Table S7: The coding potential of Lnc5q21.2

Prediction tools	Coding potential	Score	source
CPAT	Non-coding	0.000304	http://lilab.research.bcm.edu/cpat/index.php
CPC	Non-coding	-1.00148	https://cpc.gao-lab.org/
LGC	Non-coding	1.000e+00	https://ngdc.cncb.ac.cn/lgc/
PLEK	Non-coding	-2.419900	https://sourceforge.net/projects/plek/

Supplementary Table S7: siRNAs

Names	Sequences
HOXA10 siRNA#1	CCAUAGACCUGUGGCUAGATT(sense)<br>UCUAGCCACAGGUCUAUGGTT(antisense)
HOXA10 siRNA#2	CGCAGAACAUCAAAGAAGATT(sense)<br>UCUUCUUUGAUGUUCUGCGTT(antisense)
HOXA10 siRNA#3	GCAAAGAGUGGUCGGAAGATT(sense)<br>UCUUCCGACCACUCUUUGCTT(antisense)
siN6AMT1 siRNA#1	GAACUGGCAGGAGUGGAAAT(sense)<br>UUUCCACUCCUGCCAGUUC(antisense)
siN6AMT1 siRNA#2	CCUCAAGUUCACCAAGUCU(sense)<br>AGACUUGGUGAACUUGAGG(antisense)
siβ-catenin si RNA#1	CCACUAAUGUCCAGCGUUUTT(sense)<br>AAACGCUGGACAUUAGUGGTT(antisense)
siβ-catenin si RNA#2	GCCACAAGAUUACAAGAAATT(sense)<br>UUUCUUGUAAUCUUGUGGCTT(antisense)
siβ-catenin si RNA#3	GUUAUCAGAGGACUAAAUATT(sense)<br>UAUUUAGUCCUCUGAUAACTT(antisense)
